# Nanoscale Roughness in Ultra-Thick Resists by Laser-Scanning Grayscale Direct-Write Lithography and Surface Smoothing

**DOI:** 10.3390/polym18172148

**Published:** 2026-09-02

**Authors:** Giulia Malvicini, Dogukan Güçtemur, Sina Saxer, Jan Erjawetz, Helmut Schift

**Affiliations:** 1 Laboratory for Nano and Quantum Technologies, Paul Scherrer Institute (PSI), Forschungsstrasse 111, 5232 Villigen, Switzerland; helmut.schift@psi.ch; 2 nanoLab, FHNW School of Life Sciences, Hofackerstrasse 30, 4132 Muttenz, Switzerland; dogukan.guectemur@fhnw.ch (D.G.); sina.saxer@fhnw.ch (S.S.); 3 XRnanotech, Switzerland Innovation Park Innovaare, Parkstrasse 1, 5234 Villigen, Switzerland; jan.erjawetz@xrnanotech.ch

**Keywords:** grayscale lithography, direct-write lithography, ultra-thick photoresist, laser scanning confocal microscopy (LSCM), atomic force microscopy (AFM), polymer replication, thermally activated selective topography equilibration (TASTE)

## Abstract

Surface roughness at the nanometer scale limits the optical performance of reflective components for X-ray and extreme ultraviolet beam shaping. While sub-nanometer roughness can be achieved by polishing planar substrates, it remains challenging for continuous three-dimensional topographies fabricated by grayscale direct-write lithography in polymer resists. In this work, mm-long linear grayscale slopes are introduced as a calibration platform to distinguish between form, waviness, and roughness contributions. Process optimization reduces artifacts such as gray-value discretization and stitching, while replication into PMMA combined with the TASTE process enables a reduction of intrinsic roughness below 2 nm. Laser scanning confocal and atomic force microscopy are used as complementary techniques to assess surface quality across spatial scales. The results provide insight into the origin of roughness in novolak-based resists and its evolution through the fabrication chain, highlighting material limitations and paths toward smooth polymer optics with sub-nanometer roughness.

## 1. Introduction

Ultra-smooth reflective optics are a key enabling technology for beam shaping and focusing on the X-ray (<10 nm) and extreme ultraviolet (EUV, i.e., 13.5 nm) spectral ranges. At such short wavelengths, as surface height fluctuations translate into phase errors and scattering, nanometer-scale surface irregularities can measurably reduce optical throughput and degrade wavefront quality [1,2,3]. As X-ray and EUV systems move toward higher numerical aperture, tighter tolerances, and larger optical footprints, sub-nanometer to few-nanometer surface roughness is a primary limitation to optical performance [4]. Importantly, optical performance is not determined by a single topology term such as a “roughness value”: different spatial frequency bands contribute differently to scattering, imaging aberrations, and efficiency loss, making it essential to treat form, waviness, and roughness as distinct quantities (see Section 2) [1,5]. Once these components can be identified and systematic errors are mitigated, e.g., via process optimization, single roughness values, e.g., the root mean square (rms) deviation for an area could again become meaningful, not only as an absolute value for comparison of different measurement methods, but also as a relative value for determining whether an improvement can be obtained by fine-tuning fabrication methods involving copying, smoothing and coating processes.

Grayscale direct-write lithography (DWL) using intensity-modulated scanning laser exposure offers an attractive route to fabricate polymer-based three-dimensional (3D) freeform surfaces with CAD-defined topographies [6,7]. In contrast to multi-step etching and polishing workflows, grayscale exposure directly encodes a continuous height map into a single photosensitive resist layer and is compatible with large-area patterning, rapid iteration, and complex shapes. This is particularly valuable when ultra-thick resists (tens to hundreds of micrometers) are required to realize deep optical profiles [8,9]. However, the same discretized dose control that enables grayscale fabrication also introduces deterministic surface artifacts. A finite number of available gray values (GV) quantizes the intended height function and can generate step-induced waviness, while field stitching and scan strategies introduce additional periodic or quasi-periodic height modulations [10]. At the same time, intrinsic resist and process physics—including polymer microstructure, photoactive compound (PAC) distribution, and dissolution during development—set a roughness floor that can persist through subsequent replication steps [11]. When the roughness approaches the nanometer range, the sizes of resist molecules and their distribution become non-negligible and nano globular structures become visible [12]. In novolak-based resists used for grayscale DWL, these mechanisms define the intrinsic nanoscale roughness [13,14,15]. This effect is particularly relevant for ultra-thick novolak-based resists, where sufficiently low optical absorption is required to enable exposure through films approaching 100 µm thickness. This is typically associated with a reduced concentration of diazonaphthoquinone (DNQ) PACs. Although the exact molecular aggregation size and DNQ concentration of the resist investigated here are not known, polymer-chain packing, molecular aggregation, and spatial variations in PAC concentration provide plausible contributions to the intrinsic few-nanometer roughness floor.

**Figure 1:**
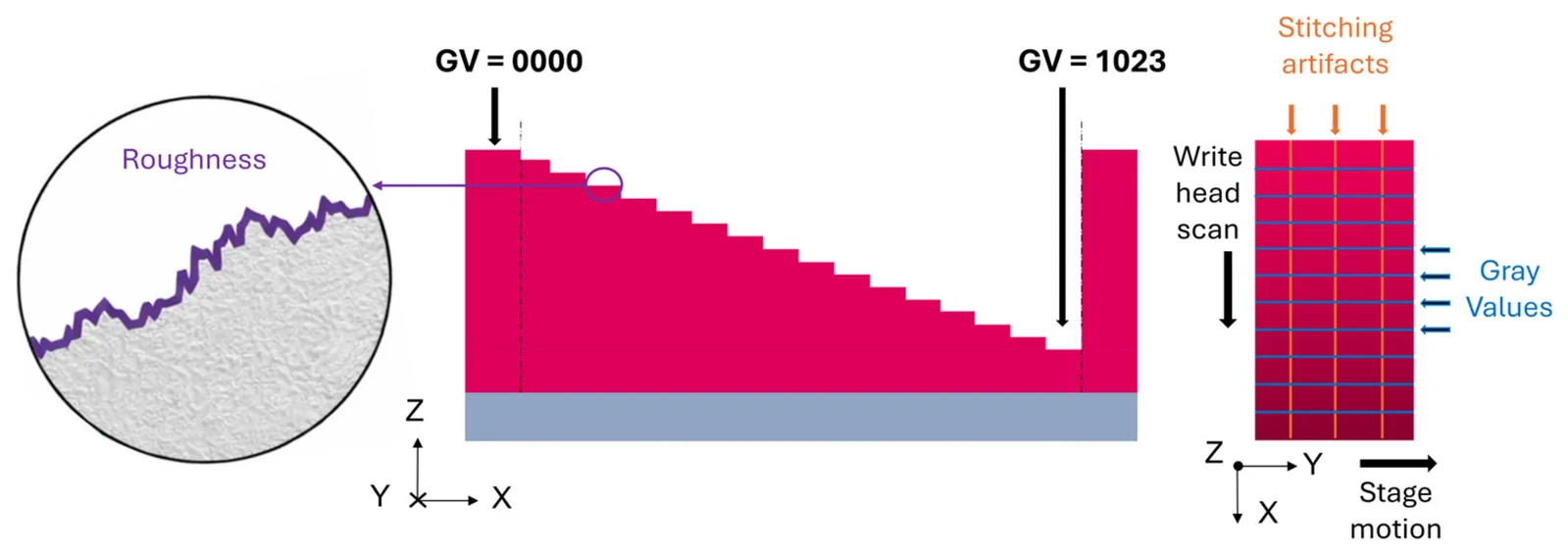
Schematic of resist topography decomposed into three components: form (linear slope), waviness (stitching artifacts and gray-values steps), and roughness. Stitching artifacts are oriented along a single direction, here y, as they originate from the stripe-based writing strategy of the DWL system. In contrast, GV discretization steps are governed by the design and therefore appear along the direction of height variation, independent of the writing direction.

A second related challenge lies in the metrology of such large features with sub-nanometer roughness. As already introduced, surface topography of extended free-form structures can be characterized by three distinct components: form, waviness, and roughness [5,16] (see Figure 1). In grayscale-fabricated 3D optics, form errors, medium-spatial-frequency waviness, and high-frequency roughness are often not clearly separated, as the analysis relies on filtering procedures that are not unique and can bias the extracted roughness values [16]. Moreover, different instruments probe different spatial bandwidths. Laser scanning confocal microscopy (LSCM) enables rapid, non-contact characterization of millimeter-scale profiles but becomes diffraction- and noise-limited at nanometer roughness levels [17]. In contrast, atomic force microscopy (AFM) provides sub-nanometer vertical resolution but is restricted in scan size and accessibility for deep or high-aspect-ratio structures. A hybrid approach is particularly effective: high-resolution methods such as AFM can be employed for roughness assessment, while techniques like LSCM allow rapid characterization of form and artifacts over larger surface areas. The combined approach is therefore required to capture the full spatial spectrum of surface features [18]. Other techniques, such as scattering-based methods, provide integrative measurements over larger surface areas. While efforts have been made to compare roughness across methods and spatial frequencies, the interpretation of results remains strongly dependent on filtering procedures and measurement conditions, complicating direct comparison between techniques [19].

In this work, we introduce extended shallow linear grayscale slopes (with ~0.5° inclination) as a controlled calibration platform for ultra-low-roughness grayscale lithography. This enables both (i) the identification and analysis of controlled fabrication of artifacts and (ii) the assessment of the bandwidth limitations inherent to each metrology method [20]. Linear slopes, with their single-gradient geometry, enable deviations to be quantified by subtracting a best-fit line (form removal) and then analyzing the residual height signal. These shallow slopes enable the visualization of discretization steps over a wide range of GVs. Such structures are also directly relevant for applications, as they approximate the shallow gradients found in reflective optical components, including inclined and concave focusing mirrors operating under grazing-incidence conditions over millimeter-scale distances. This geometry allows deterministic contributions, such as GV quantization steps, stitching artifacts, and scanner-induced ripples, to be readily identified as waviness, while enabling localized AFM measurements of stochastic roughness in accessible regions. Beyond metrology, linear slopes provide a convenient platform for directly comparing how surface features evolve throughout the complete fabrication chain, from the resist master to replicated polymer structures.

The scope of this paper is threefold: (i) demonstrating the achievement of nm roughness in polymer surfaces manufactured with 3D DWL by eliminating systematic sources of roughness in DWL exposure and development, (ii) using and qualifying LSCM and AFM as techniques to assess the quality of measurement, and (iii) establishing linear slopes as a practical test structure for separating and quantifying form, waviness, and roughness in ultra-thick grayscale lithography.

## 2. Materials and Methods

### 2.1. Grayscale Lithography Fabrication Process

Grayscale lithography was performed using a DWL 66^+^ direct laser writer from Heidelberg Instruments GmbH, Heidelberg, Germany, operating at a wavelength of 405 nm. The system employs a scanning laser beam with fast intensity modulation via an acousto-optical modulator, enabling spatially resolved dose control and grayscale exposure. The maximum number of available GVs is 1024 (10-bit gray-level dynamic range), and the minimum pixel size is 50 nm. For the DWL 66^+^ installed at PSI in 2020, this was a big improvement over the DWL 66FS with 128 GVs (4-bit) and the µMLA Maskless Aligner with 256 GVs (8-bit) [21]. Current DWL 66^+^ configurations support substantially higher grayscale resolution, up to maximum number of 65,536 GVs (16-bit). For shallow slopes, e.g., 10 mm long linear slopes in 100 µm thick resists, and 1024 GVs, however, this results in 9.7 µm wide steps that are larger than the typical sizes of 0.8 µm or 1.3 µm wide laser beams for the write modes (WM) I (4 mm focal length) and III (10 mm focal length), respectively. This would result in steps that would be separated by horizontal terraces, while smaller step sizes would be smoothened out by the overlapping laser beam.

For all structures discussed in this work, the coordinate system is defined such that the x-direction corresponds to the slope-gradient direction and to the fast laser-scanning direction, whereas the y-direction corresponds to the orthogonal substrate-stage translation direction. Accordingly, the grayscale height variation is encoded along x. The DWL system operates by scanning the laser beam using an acousto-optic deflector along the x-direction over a finite stripe width while the substrate stage moves in the orthogonal y-direction. As a result, the exposure is segmented into adjacent stripes with well-defined boundaries in one direction only. Stitching artifacts therefore arise at the interfaces between neighboring stripes, while along the scan direction the exposure is continuous. To reduce the effect of stripe-to-stripe stitching, multi-pass exposure strategies were employed in which the same nominal pattern is distributed over N laterally shifted sub-exposures (called ‘N-over’ N). In this work, CI-over N denotes an advanced strategy offered by the Heidelberg Instruments DWL proprietary data preparation software, that overlaps N subfields with a modified intensity distribution along the stripe, thereby smoothing the intensity transition between adjacent subfields. The practical effect is a reduction of the effective stitching height and stitching periodicity by a factor N. When using the WM III, the nominal stripe width is 300 µm, which reduces to 30 µm with CI-over 10 and 7.5 µm with CI-over 40.

For comparison with the DWL 66+, exposures were performed at RAITH, Best, The Netherlands, using the RAITH PICOMASTER 200 at 405 nm laser wavelength and the same resist process conditions (Section 3.5). This DWL tool allows modulation of the laser beam intensity at 4096 GVs (12-bit). It employs a stationary beam, with respect to the optical axis, and mechanically decoupled scan (Y) and step (X) stages. This approach inherently eliminates stitching, allowing for uniform, stitch-free fabrication regardless of design size without requiring a multi-pass strategy for stitching mitigation.

Ultra-thick photoresist layers were fabricated using mr-P 22G XP, a novel novolak-based positive-tone resist from micro resist technology GmbH, Berlin, Germany, which was designed for grayscale lithography for thicknesses over 100 µm, by reducing the PAC concentration that would limit absorption. Novolak is known for its relatively large molecules and occurs as agglomerates, with a size of a few nm [22]. Substrates were cleaned sequentially with isopropanol, acetone, and oxygen plasma. An adhesion promoter (Surpass 4000 from DisChem, Ridgway, PA, USA) was applied by spin-coating to improve film uniformity and repeatability, particularly for resist thicknesses approaching 100 µm.

To minimize bubble formation, which results from the resist curing chemistry and is particularly prone to thick resists, the resist was degassed in a glass beaker for two hours prior to spin coating. A constant resist volume of 5 mL was dispensed onto 100 mm wafers, followed by spin coating at 1000 rpm with an acceleration of 2000 rpm·s^−1^ for 4 s. Soft baking was performed using a temperature ramp from 30 °C to 90 °C with intermediate plateaus at 50 °C and 80 °C. After exposure, samples were developed in AZ726 MIF from MicroChemicals GmbH, Ulm, Germany, for 60 min without post-exposure bake. Subsequently, the developed resist was treated at 90 °C for 1 min to induce surface smoothening around the anticipated glass transition temperature T_g_ of the novolak, which contains DNQ as PAC [23]. The 60 min development time was selected for the approximately 100 µm thick grayscale resists. To assess the influence of the prolonged development step independently from exposure, unexposed mr-P 22G XP regions subjected to the same development conditions were additionally characterized by AFM and compared with spin-coated, undeveloped resist. The corresponding measurements are reported in the Supplementary Materials.

### 2.2. Pattern Transfer Workflow

Linear slope masters were fabricated in mr-P 22G XP (at 100 µm thickness) by grayscale direct-write lithography as described above. Prior to replication, an anti-sticking layer (ASL) was deposited on the resist master using vapor-phase silanization [24]. The silane precursor was introduced into a vacuum chamber containing the wafers, forming a monolayer of densely packed fluoroalkyl chains on the resist surface. Only 5 milliliters of silane were required, resulting in a deposition of a 1–2 nm thick ASL.

Replication of the novolak originals was considered for several purposes, including the multiplication of the initial master, form reversal, transfer into more stable materials, and the potential for surface smoothing using materials with smaller molecular size. However, these advantages can only be realized if the replication process does not significantly increase surface roughness relative to the original.

Subsequently, the UV-curable polymer GMN PS90 from Optool AB, Veberöd, Sweden, with a Young’s modulus of 114.3 MPa and low shrinkage of ≤0.1%, was cast directly onto the treated master [25]. A Borofloat glass wafer, previously spin-coated with OrmoPrime 20 adhesion promoter, was placed on top of the liquid resin, forming a sandwich structure. The assembly was then exposed in a UV flood exposure system at 356 nm with a dose of 1500 mJ·cm^−2^ to fully cure the GMN PS90.

After curing, the glass wafer with the attached GMN PS90 replica was delaminated from the resist master. The resulting rigid polymer replica required no additional processing and was used directly as an imprint stamp.

The thermoplastic material used for replication and subsequent surface smoothing was a poly methyl methacrylate (PMMA) foil (Plexiglas Film 0F058, Evonik, Essen, Germany). PMMA is a material with linear chains composed of small monomeric elements that have proven to have a high resolution in both electron beam (EBL) and nanoimprint (NIL) lithography. According to the manufacturer’s specifications, the material exhibits a T_g_ of ~109 °C [26], which is consistent with bulk, high-purity PMMA. This thermoplastic behavior is essential for enabling controlled thermal processing steps. Thermal NIL was performed at 160 °C, well above T_g_, to ensure sufficient polymer flow for faithful replication of the master structure. For the replication of a nanometer-scale reference sapphire sample (Al_2_O_3_ STEP substrate) from Shinkosha Co., Ltd., Yokohama, Japan, via thermal imprint, no ASL was applied to avoid smearing out of the 0.26 nm high and 0.20 µm long terraces by the 1 nm long alkyl chains.

For PMMA, thermal imprint was applied to ensure replication of the height gradient. Both replication methods were optimized to preserve slope linearity and surface smoothness and minimize defects.

The TASTE (thermally activated selective topography equilibration) process was employed to further refine and transfer the slope profiles [27,28]. TASTE is used here as a depth-confined reduction of T_g_ by vacuum ultraviolet (VUV) light by breaking the chains of a linear polymer such as PMMA. By reducing its molecular weight M_w_ and T_g_ locally, it enables the thermal reflow of a surface at a temperature slightly below the T_g_ of the pristine material, while preserving the overall shape of a 3D structure [27,29]. This enables, e.g., the smoothing of waviness or roughness below 200 nm laterally or vertically regarding size while preserving the shape of several µm in size, thus reducing surface roughness to ~1 nm. After initial replication, the PMMA samples were exposed to 172 nm VUV radiation in air at room temperature for 30 s, corresponding to a total dose of approximately 465 mJ·cm^−2^, with the sample positioned approximately 10 mm opposite from the lamp. Immediately after VUV exposure, the samples were thermally treated at 105 °C for 1 min.

The validity of the TASTE process for smoothening of nanometer-sized surface roughness will be discussed in Section 3.3, and its effect will be discussed in Section 4.4.

### 2.3. Surface Characterization

LSCM (VK-X3100, Keyence Corp., Osaka, Japan) was used as a non-contact technique for large-area and deep-profile characterization of the three-dimensional surface profile of the fabricated slopes over millimeter-scale lengths. AFM (Dimension Icon from Bruker Switzerland AG, Fällanden, Switzerland) was employed to measure local surface roughness with sub-nanometer resolution on accessible regions. AFM measurements were complementary to LSCM and were essential, as they provide direct access to the intrinsic roughness regime, in the order of nm or sub-nanometer, which cannot be measured by LSCM. Measurements were conducted in non-contact mode using probes with a tip height of 4 µm, a cantilever length of 125 μm, and a width of 30 µm (NCH-10, Nanoworld, Neuchâtel, Switzerland). The resonance frequency was 320 kHz and force constant was 42 N/m.

#### 2.3.1. LSCM Capabilities and Limitations

LSCM is a well-suited tool for 3D surface topographies in the micrometer range. For structures that reach mm size on one axis, LSCM can provide fast results for assessing the main shape of the 3D structure. The wavelength of 404 nm provides high resolution and different types of focusing lens objectives allow inspecting the shape on different spatial scales. According to the specifications, the system offers a 0.01 nm resolution in the z-dimension, but roughness measurements also depend on the lateral resolution, which is fundamentally limited by diffraction (see Formula (1)):

(1) $$\delta_{x}=\frac{0.61\lambda }{N_{A}}$$

which results in $\delta_{x}$~100 nm for the highest magnification (where λ is the wavelength and N_A_ is the numerical aperture). In addition, mechanical vibrations from the environment (e.g., air ventilation and floor vibrations) introduce background noise, manifesting as quasi-periodic features. This effect is evident in both exposed and unexposed regions, indicating that the observed waviness originates from the measurement system rather than from the fabricated structures.

LSCM enables reliable reconstruction of two-dimensional height profiles over millimeter-scale lengths due to its non-contact operation and large vertical scanning range. In this work, extended slope shapes with dimensions of approximately 10 × 0.3 mm^2^ were measured using a ×20 objective, requiring the acquisition and stitching of multiple adjacent fields of view to cover the full slope length. Stitching-related artifacts coming from the measurement technique are therefore observable in the reconstructed images. The use of higher-magnification objectives (e.g., ×50) was also pursued, even if the total acquisition time increased.

Surface roughness measurements were performed using a x150 objective to exploit the highest available vertical resolution of the LSCM system. Increasing the objective magnification improves the axial and lateral resolution of the reconstructed height data, at the expense of a reduced field of view, longer acquisition times, and increased sensitivity to environmental noise and mechanical vibrations.

Table 1 summarizes the three main classes of contributions affecting the LSCM and AFM measurements: fabrication-related artifacts, intrinsic surface roughness, and measurement noise.

At ×150 magnification, the acquired image size was approximately 95 × 267 µm^2^. Although this small field of view would require extensive stitching to cover larger areas, e.g., of 10 × 10 mm^2^, only a single image was intentionally used for roughness analysis to avoid additional roughness contributions or artifacts that could be introduced by image stitching. 

Despite its high vertical resolution, the limited lateral bandwidth of LSCM restricts its ability to resolve high-frequency surface features. Consequently, roughness values at the nanometer scale may include contributions from unresolved structures and measurement noise and should therefore be interpreted with caution.

#### 2.3.2. AFM Capabilities and Limitations

The main limitation of AFM measurements in this work arises from the large depth of the resist structures, which can reach up to ~100 µm. AFM provides high vertical resolution and is therefore well suited for quantitative roughness analysis on shallow or moderately deep structures. However, its applicability to deep 3D topographies is intrinsically limited by the finite geometry of the AFM tip and by the restricted vertical travel range of the scanner [18]. Accessing the bottom regions of deep features becomes increasingly challenging as the structure depth approaches or exceeds the maximum z-range of the AFM or the lateral dimensions of the structure are sufficiently large to accommodate the cantilever. To address this limitation, roughness measurements were performed on slope structures either designed to be sufficiently wide to ensure safe tip access or surrounded by high-dose frames that make specific depths (~20–60 µm) of a slope accessible.

AFM topography data were analyzed using WSxM 5.0 Develop software [30], applying preprocessing steps, such as first-order plane removal and line-offset correction, only where required to compensate for scan-line offsets, prior to extracting rms roughness (S_q_).

## 3. Results

With the current DWL 66^+^ system, the smoothest extended slopes were obtained using GV1024 combined with a CI-over 40 multi-pass exposure strategy. In this configuration, the nominal grayscale step length in the x-direction was reduced to 9.7 µm, while the effective stitching width in the y-direction decreased to 7.5 µm. In contrast, GV256 with CI-over 10 was intentionally employed as a diagnostic condition to directly resolve quantization-induced waviness, since the larger nominal step length (~39 µm over a 10 mm slope) remains clearly distinguishable from smoothing effects.

Linear slopes with a total length of L = 10 mm and width W = 300 µm were fabricated in 100 µm thick mr-P 22G XP using grayscale direct-write lithography. The slope height was adjusted by varying the exposure power. Under the optimized GV1024 condition, the reduced step length, combined with multi-pass overlap exposure and resist reflow, sufficiently suppressed deterministic artifacts such that they were no longer detectable with the current measurement techniques.

### 3.1. Non-Optimized Exposure

To identify and distinguish the dominant deterministic artifacts in grayscale DWL, two non-optimized exposure conditions were intentionally selected to isolate the effects of gray-value discretization and field stitching, respectively.

Firstly, the influence of gray-value quantization was investigated by reducing the number of GVs to 256 (GV256), while applying a high-overlap multi-pass strategy (CI-over 40). The resulting nominal step length (39 µm for a 10 mm slope) is large in comparison to smoothing effects to enable the resolution of quantization-induced waviness directly. Under these conditions, residual periodic structures can be attributed predominantly to discretization of the grayscale dose.

Secondly, stitching artifacts were isolated by maintaining the maximum GV resolution (GV1024) while reducing the overlap to CI-over 10. In this configuration, the effective stitching periodicity remains visible. As described and shown later in the section, the periodic height modulations with a characteristic spacing correspond to the reduced overlap distance (30 µm) along the writing direction.

By separating these two contributions experimentally, it becomes possible to distinguish between GV discretization-induced waviness and periodic stitching-related artifacts, both of which can be attributed to an increase in roughness. By measuring on the terraces between steps or between stitching artifacts, the material-related surface roughness can be determined.

**Figure 2:**
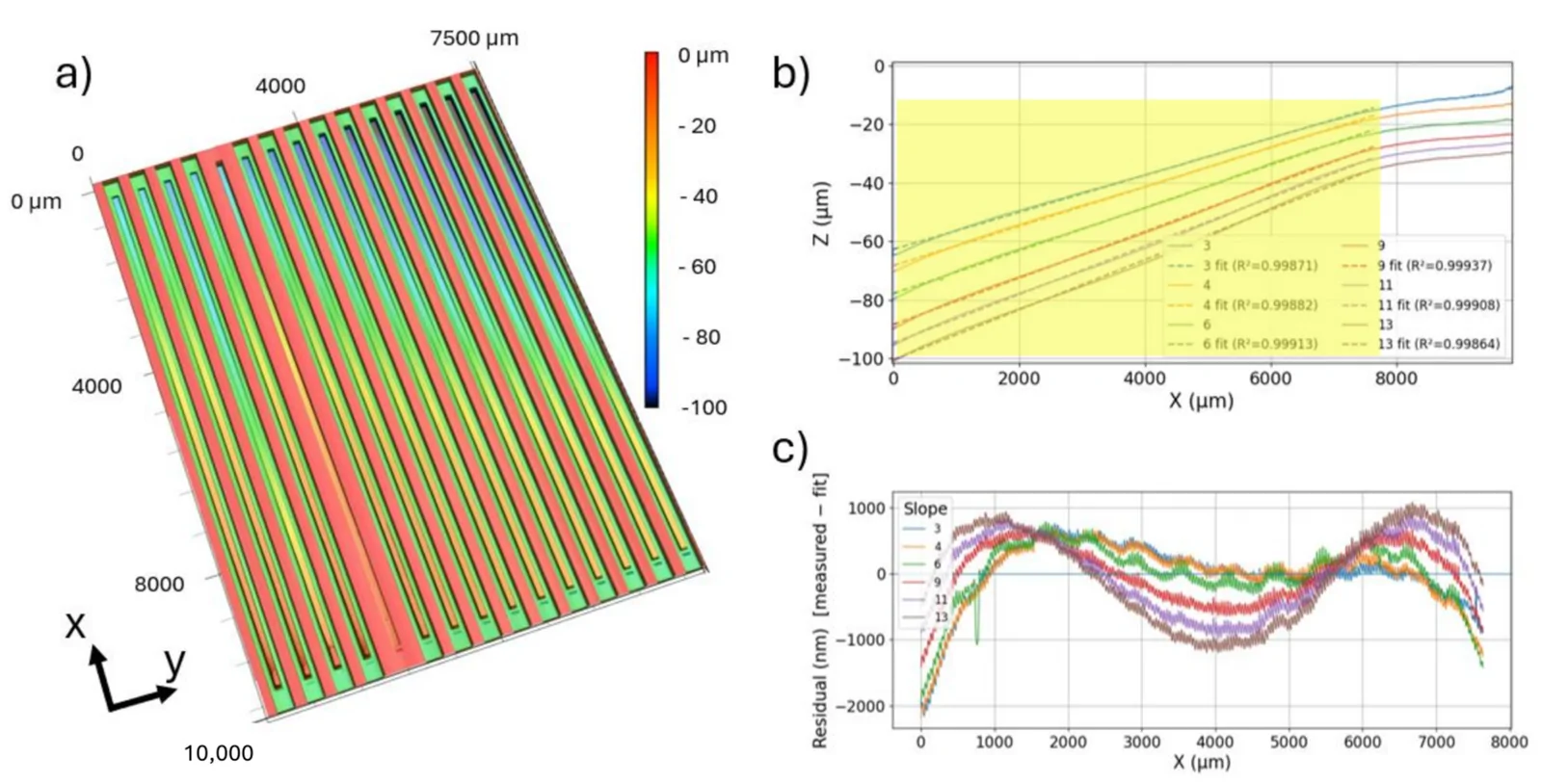
(**a**) LSCM measurements of 15 linear slopes exposed at different laser powers with slopes descending from top to the bottom of the figure. (**b**) Representative 2D profiles of slopes no. 3, 4, 6, 9, 11, and 13, from left to right. (**c**) Residuals after linear form removal. The structure was written with WM III, 256 GVs and CI-over 40.

Figure 2a shows 15 slopes fabricated using WM III, 256 GVs and CI-over 40, at different exposure powers (increasing from slopes 1 to 15), which determine the resulting depth gradient.

The x-direction corresponds to the slope direction (uphill) while the y-direction is perpendicular to the slopes. Slope 5 from the left appears different due to the absence of a surrounding frame; however, it exhibits the same behavior as the other structures. Figure 2b presents six selected slopes, identified by the numbers in the legend and corresponding to the structures shown in Figure 2a, where slope 1 is located on the left. All slopes exhibit a nonlinear transition close to the upper resist surface, followed by a quasi-linear region. To exclude the nonlinear transition from the quantitative form analysis, the linear fits were performed over the interval x = 0–7500 µm, with x = 0 corresponding to the deepest point of the slope and x = 7500 µm to the most superficial point included in the fit. The fitting range is now highlighted in the yellow area in Figure 2b.

For the representative slopes 3, 4, 6, 9, 11, and 13, exposed at 63, 70, 83, 105, 115, and 130 mW, respectively, and a filter transmitting only 12.5% of the total power, the measured height differences over the fitted 7.5 mm interval were 51, 52, 57, 62, 62, and 64 µm. These correspond to average gradients of 6.80, 6.93, 7.60, 8.27, 8.27, and 8.53 µm/mm and inclination angles of 0.390°, 0.397°, 0.435°, 0.474°, 0.474°, and 0.489°, respectively. As expected, increasing exposure power generally results in a larger developed depth.

The measured profiles are shown together with their respective linear fits. Figure 2c displays the residual error, defined as the difference between the measured profile and the corresponding linear fit. The initial deviation is attributed to dose threshold effects and nonlinear resist response near the surface. Beyond this region, the slope follows the intended geometry, exhibiting a form deviation below 2 µm over a length of 10 mm and a depth of 100 µm.

**Figure 3:**
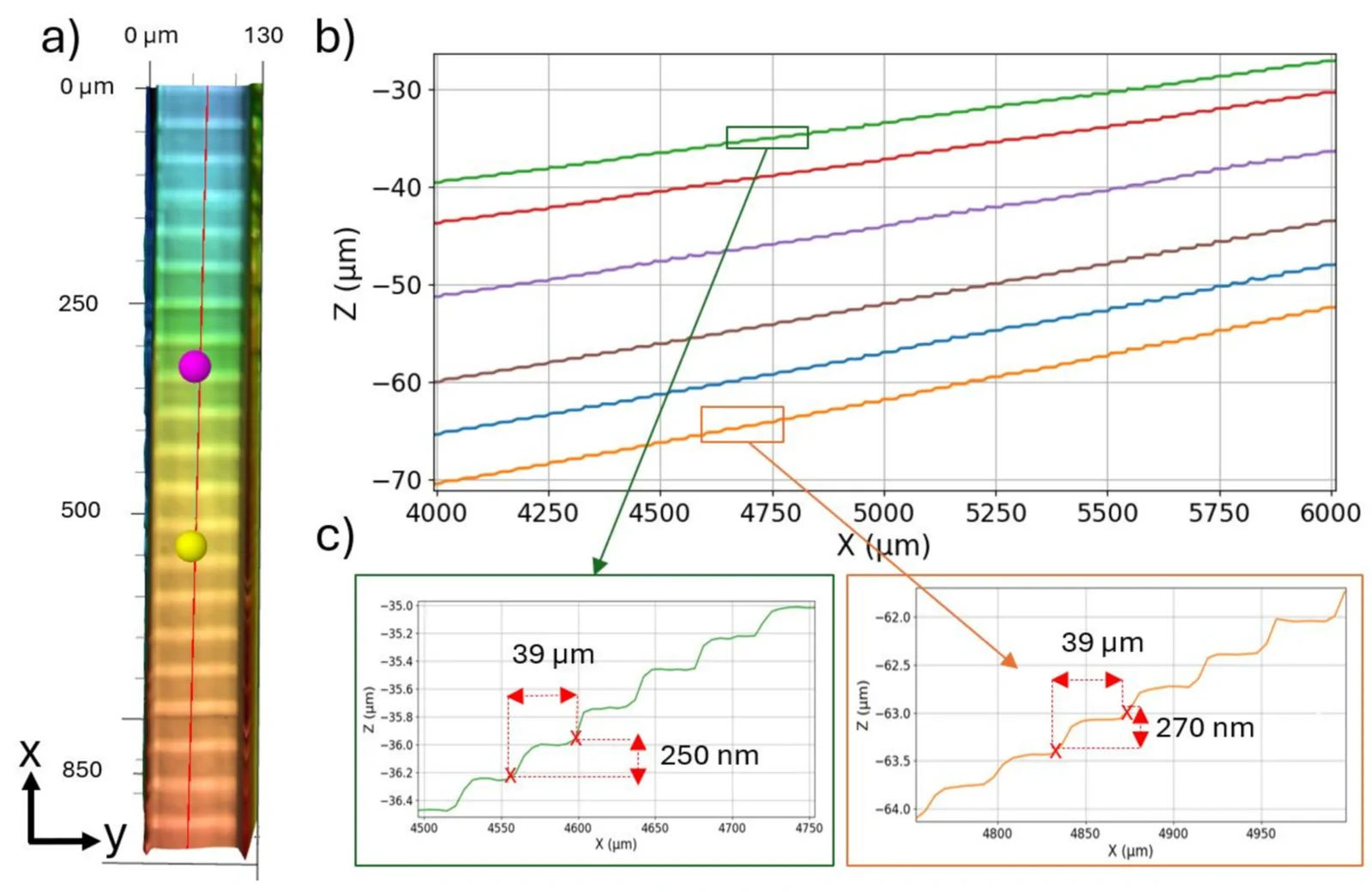
(**a**) LSCM measurements of linear slope fabricated in mr-P 22G XP on the DWL 66^+^ using WM III, 256 GVs and CI-over 40. The pink and yellow dots indicate two points along the line used to extract the profile. (**b**) 2D profile of linear slopes with different depths. (**c**) The lateral step spacing remains constant at ~39 μm, consistent with the imposed discretization, while the step height varies slightly due to the local change in slope angle (smaller at the beginning, larger toward the end).

When fabricated using 256 GVs and a 40-pass multi-exposure strategy with CI-over for WM III, the writing field and thus the periodicity of stitching is reduced from 300 µm to 30 µm, and deterministic waviness dominates the residual profile. For a 10 mm slope discretized into 256 levels, the nominal lateral discretization length is approximately 39 µm. As shown in Figure 3, LSCM measurements reveal step-like features with a measured spacing of 39 µm between horizontal terraces, in excellent agreement with the imposed gray-value discretization.

This confirms that these structures arise from design-imposed quantization rather than stochastic process noise. The rounding of the steps originates from the 1.2 µm wide WM III beam, which smooths sharp corners while leaving the terraces unaffected. The heights are 250 nm and 270 nm, which is in accordance with a ~60 µm high linear slope.

**Figure 4:**
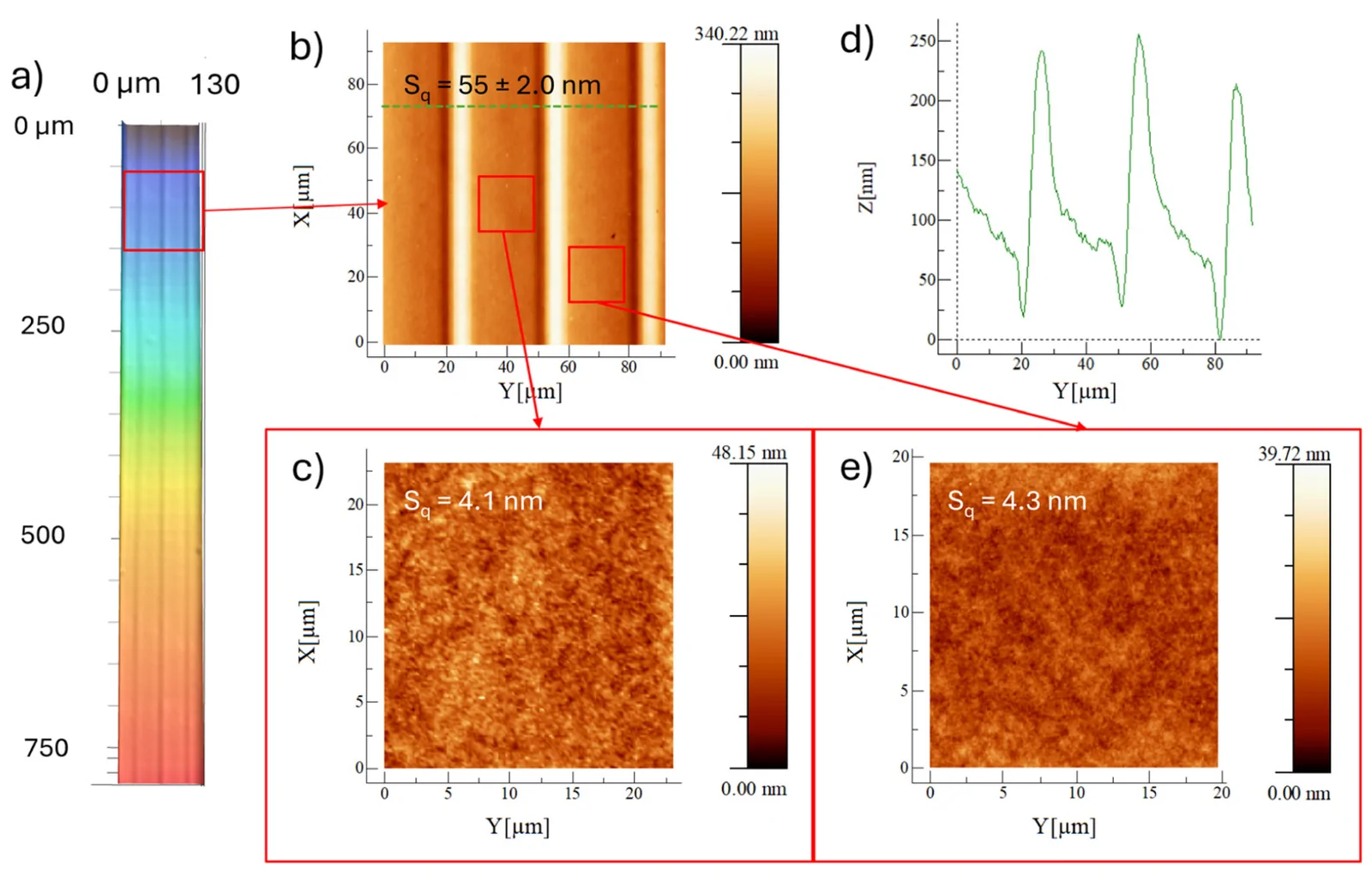
Exposure artifacts on a linear slope fabricated by grayscale direct-write lithography (DWL). (**a**) LSCM measurements of a linear slope fabricated in mr-P 22G XP on the DWL 66^+^ using WM III, 1024 GVs and CI-over 10. (**b**) AFM image of the slope, the position is indicated in the LSCM image (**a**), with a measured total RMS roughness of 55 ± 2.0 nm, which includes both the step and intrinsic roughness; (**d**) 2D profile perpendicular to the slopes, thus scanning over the stitching errors, represented by ~170 nm high steps, showing peak-and-valley behavior at the borders of the writing fields; (**c**,**e**) AFM measurements in between writing fields showing roughness of S_q_ = 4.1 and S_q_ = 4.3 nm, respectively.

In addition, periodic stitching artifacts with characteristic lateral spacing of ~30 µm are observed along the slope direction (Figure 4a), which are in accordance with the 30 µm staggered multi-pass exposure of the WM III 300 µm overlapping stripes. They originate from stripe-based writing architecture rather than insufficient dose averaging and are, due to the lower doses in each stripe and the smoother transition between stripes, still visible in an N-over 10 exposure. After linear form removal, the residual waviness amplitude is ~200 nm peak-to-valley, corresponding to S_q_ = 55 ± 2.0 nm measured by AFM over a 95 × 95 µm^2^ scan area.

AFM measurements performed locally on step regions (Figure 4d,e) that are present as terraces and reveal that the high-frequency roughness component remains significantly smaller, with S_q_ = 4.1 ± 0.2 nm. By choosing the scan field of 20 × 20 µm^2^, measurements can be confined to areas without stitching artifacts in the x-direction. At the same time, the scan field extends over 2 GV steps in the y-direction that are 9.7 µm long and 30 nm high. These steps are smoothened out. This confirms that the dominant contribution for high S_q_ in the GV1024 case is stitching artifacts rather than material-intrinsic nanoscale roughness.

In the CI-over 10 condition, artifact amplitudes were reduced compared with single-pass exposure (CI-over 1) but remained intentionally resolvable, making this condition useful for diagnosing the origin of waviness. The fully optimized condition employed GV1024 together with high-overlap exposure (CI-over 40), in which deterministic features were largely suppressed.

### 3.2. Effect of Grayscale Optimization via GV1024 and High Multi-Pass

**Figure 5:**
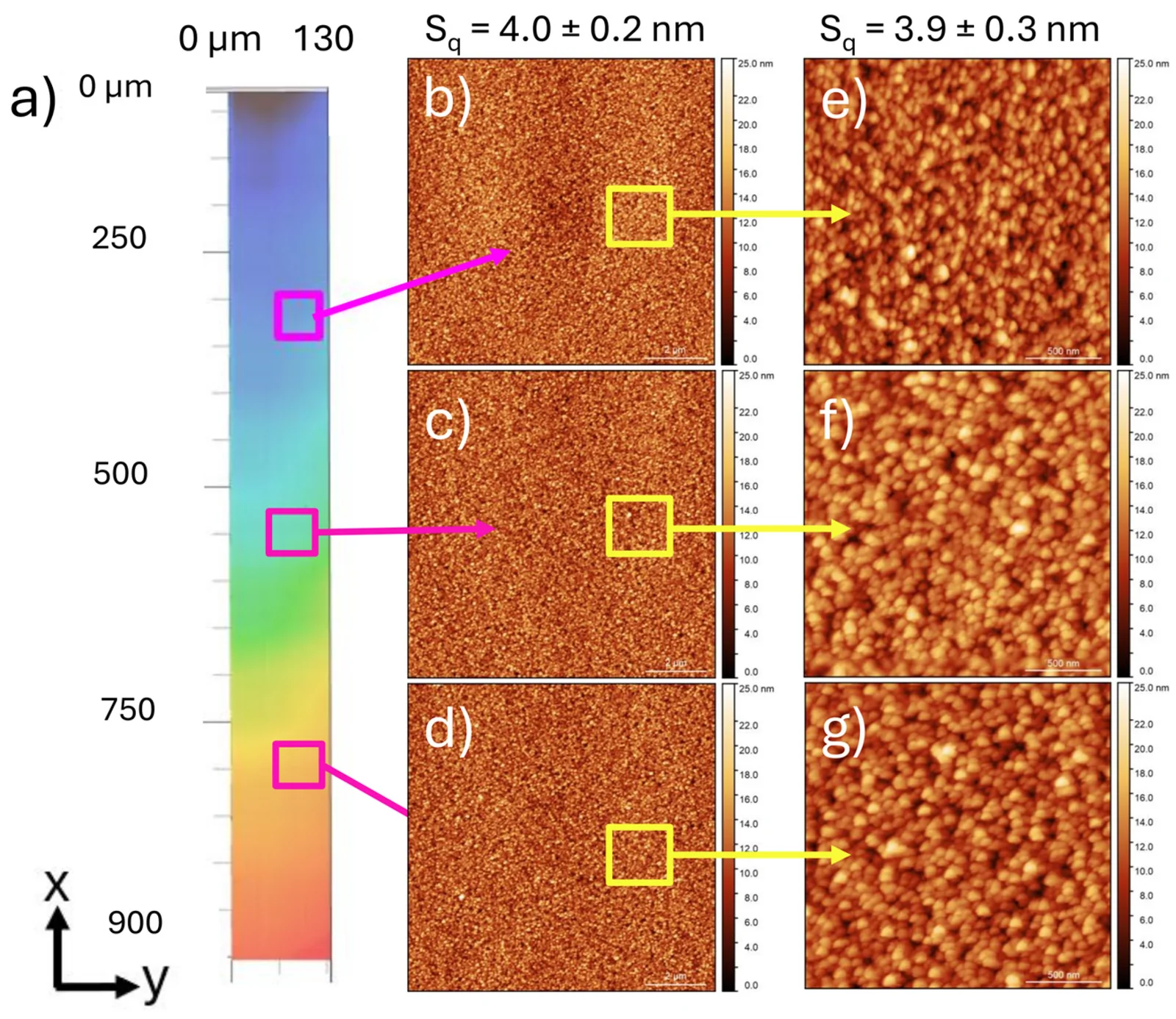
(**a**) LSCM measurement of the linear slope. (**b**–**d**) AFM topographic images acquired at three different positions along the slope over scan areas of 10 × 10 µm^2^. The average surface roughness obtained from these measurements is 4.0 ± 0.2 nm. (**e**–**g**) Corresponding 2 × 2 µm^2^ AFM images acquired within the 10 × 10 µm^2^ regions shown in (**b**–**d**), respectively, yielding an average roughness of 3.9 ± 0.3 nm.

In the optimized condition employing GV1024 and CI-over 40 multi-pass exposure, as shown in Figure 5a, quantization steps in the x- (steps) and y-direction (stitching) are no longer clearly resolved along the central part of the slopes. The strong suppression of step-like waviness over these length scales confirms that increasing the number of gray values effectively reduces medium-spatial-scale artifacts; as already mentioned in the introduction to Section 3, the nominal quantization for GV1024 would theoretically produce steps of 9.7 µm length and ~58.6 nm height. In this optimized condition, the quantization steps along x and stitching-related modulation along y are no longer clearly resolved after development and resist reflow. The nominal GV1024 discretization corresponds to a lateral pitch of approximately 9.7 µm and, for the investigated slope, to a nominal height increment of approximately 58.6 nm.

The disappearance of these features cannot be attributed to the finite laser-beam width alone. The WM III beam width of approximately 1.2–1.3 µm is considerably smaller than the 9.7 µm GV pitch and therefore primarily rounds the boundaries between adjacent grayscale levels rather than fully averaging neighboring terraces. Instead, the final suppression of the deterministic topography results from the combined fabrication sequence. The CI-over 40 exposure strategy distributes the dose over multiple laterally shifted passes and reduces local exposure discontinuities, particularly at stripe boundaries. The subsequent development step introduces additional profile rounding through the nonlinear dose-to-depth response of the positive-tone resist. Finally, thermal reflow of the developed mr-P 22G XP at 90 °C for 1 min further levels residual surface-height variations.

AFM measurements performed before resist reflow, presented in Figure S3, show that GV-related topographic modulation remains detectable prior to the thermal treatment. In contrast, after reflow, these periodic features are no longer resolved under the optimized fabrication condition. This comparison indicates that optical overlap and multi-pass averaging reduce the amplitude of the deterministic features, while thermal reflow provides the final smoothing of the residual GV-induced topography. AFM measurements performed at different positions along the slope provide access to the high-frequency roughness regime. For 10 × 10 µm^2^ scan areas, the developed resist exhibits an average roughness in nm, while the corresponding 2 × 2 µm^2^ regions yield the nm values shown in Figure 5b–g.

Importantly, neither systematic dependence of roughness on the slope angle or position along the slope is observed, nor on the exact positioning of the scan field. This indicates that the nanoscale roughness is primarily governed by intrinsic material properties and stochastic development processes rather than grayscale exposure parameters. Grayscale optimization therefore primarily reduces deterministic waviness, while the intrinsic roughness floor remains approximately constant. This interpretation is further supported by cross-platform measurements performed on PICOMASTER-exposed samples (Section 3.5), a DWL, where the absence of grayscale-induced waviness does not result in a reduction of nanoscale roughness. This increase in the number of GVs is now also available in the latest version of the DWL 66^+^. The consistent roughness values across fundamentally different exposure systems confirm that this roughness represents an intrinsic material limit rather than a tool-specific artifact.

### 3.3. Validation of the TASTE Smoothing Mechanism

Reflow techniques have been used for a long time and use the ability of thermoplastic polymers to flow when heated above their T_g_ and assume a shape governed by surface energy, pinning and volume conservation, as occurs when wetting surfaces with liquids. This has been used for reducing line edge roughness (LER) that defines the lateral size variation of lithographically generated lines in integrated chip (IC) manufacturing. The development of the so-called self-perfection by the liquefaction (SPEL) process was instrumental, in which roughness of line boundaries of an entire line that is pinned to the substrate was smoothened out by thermal reflow [31]. Using this method, S.Y. Chou and Q. Xia reduced the 3sigma line-edge roughness of a 70 nm wide chromium grating lines from 8.4 nm to less than 1.5 nm (i.e., after transfer of the lithographically defined resist pattern into an underlying chrome layer by etching). The reflow techniques are the same as those used for the development of spherical microlens arrays, originally proposed by Popovic in [32]. Here, melting of a polymer leads to instantaneous, fast conversion of a cylinder-like resist structure with vertical sidewalls into a spherical lens with a specific size and curvature. The process does not preserve any 3D topography of a resist structure but instead creates a rounded one with minimum surface energy, while applying a set of boundary conditions to guide the flow of the molten material into the desired geometry before solidification. In [33], an average microlens surface roughness (R_a_) of 3.7 nm was measured on a 1 × 1 μm^2^ surface area on the top of a PDMS microlens. SPR 220 (Kayaku Advanced Materials, Tokyo, Japan) was used as the photoresist for reflow, which is novolak-based. The process was further developed into an approach to reduce the surface roughness on PMMA structures by using grayscale EBL [34]. For staircase structures with steps of equal lateral and vertical dimensions, reflow could, however, create slopes only if it used predefined 3D pinning structures or created local inhomogeneities in the material. Then it can reproduce the stepped slope, but without stairs [35].

Therefore, TASTE, in contrast to reflow of an entire polymer structure that is heated to induce thermoplastic melting, only smoothens out surface undulations (sub µm gratings, staircase steps, scanning increments, stitching artifacts, and roughness from serial lithographic scanning processes) by locally softening polymers above their T_g_, while other parts, e.g., the bulk below a surface skin area, remain unaffected. This is either possible by locally modifying the polymer, e.g., by reducing its T_g_ thereby increasing its ability to flow, or by selective heating. The latter case is possible if only the surface is heated (e.g., by irradiative heat transfer) by placing the polymer sample on a colder holder while facing a heat source. In previous experiments with EBL exposure, the weight-averaged molecular weight M_w_ of the pristine, moderate M_w_ PMMA was reduced by exposure with 172 nm light from 91.2 kg·mol^−1^ to below 9 kg·mol^−1^ (averaged molecular weight M_n_ from 40 kg·mol^−1^ to 4 kg·mol^−1^), measured by quantitative DSC analysis (differential scanning calorimetry), which corresponded to a decrease of the T_g_ from 122 °C to 90 °C [27]. The surface-selective process using VUV exposure and polymer films with a lower T_g_, as used in this research, is described in [29].

Although the reflow temperatures with the simple setup from [29] cannot be as precise as possible with the setup for thermal-radiation-induced local reflow (TRILR) described in [34], parameters such as time and distance (i.e., temperature) can be tuned over a wide range, with the aim of achieving a temperature above the T_g_ of the modified but below the T_g_ of the unexposed PMMA. Furthermore, since the aim of the reflow is a smooth surface (i.e., one of minimum surface energy), a reflow will typically lead to a stable equilibrium of minimum surface roughness and therefore longer process times are tolerated. Therefore, we can assume that the M_w_ values of the films used in this research, with a T_g_ of 107 °C, are similar to those in [27].

To demonstrate that the TASTE process selectively smooths high-frequency features, a nanometer-scale reference sapphire sample with ~0.3 nm atomic-scale surface step structures was first characterized by AFM and subsequently replicated into PMMA via thermal imprint. Such single-crystal α-Al_2_O_3_ templates have been previously used to demonstrate sub-nanometer fidelity replication into PMMA by thermal NIL with 0.26 nm high straight steps and 600 nm wide terraces [36,37]. These results were reproduced by imprinting sapphire molds with 0.26 nm high straight steps and 200 nm wide terraces, which were subsequently smoothened by selective thermal reflow. For this, the resulting PMMA replica was then divided into two regions: one subjected to thermal reflow using the TASTE process, and one left untreated.

**Figure 6:**
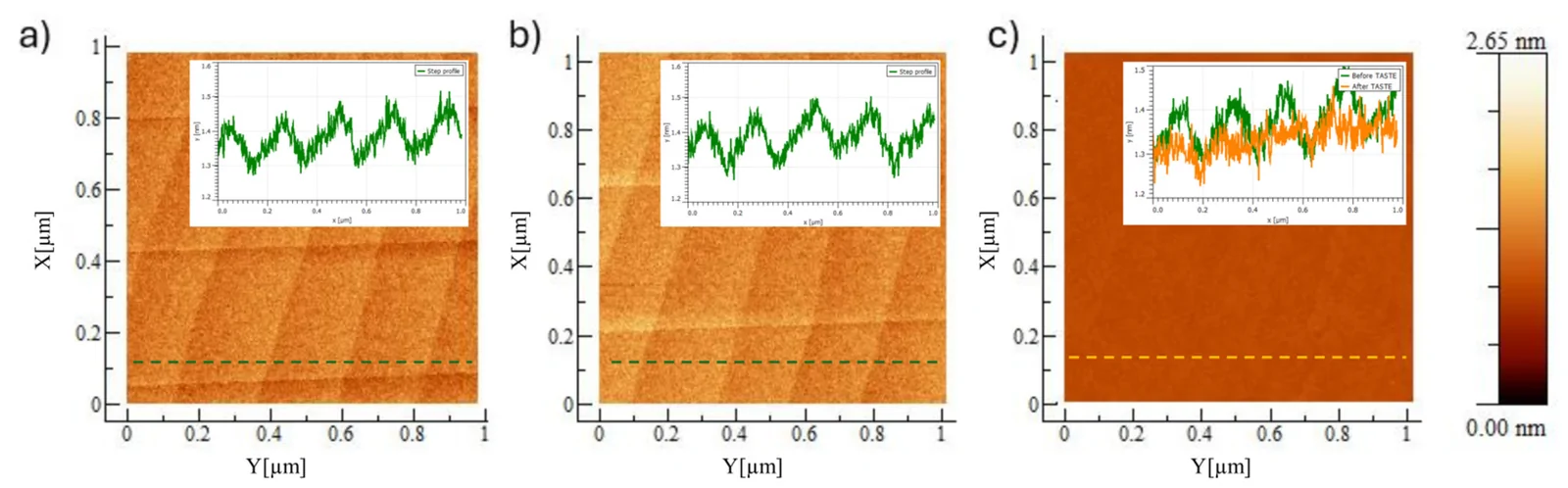
AFM measurement of single-crystal sapphire template with atomic-scale 0.26 nm high and 0.2 µm wide surface steps over 1 × 1 µm^2^ area. Inlet: 2D profile along dotted line; (**a**) original sapphire sample. (**b**) imprint in PMMA; (**c**) after TASTE process. The inlet in part (**c**) shows a comparison of the 2D profile before (green) and after (orange) TASTE. S_q_ values are indicated in the figures.

AFM characterizations of the mold, the replicated sample, and the post-processed sample are shown in Figure 6. Figure 6a presents a 1 × 1 µm^2^ scan of the original sapphire surface, revealing the step-like topography, resulting in a sub-nanometer roughness of S_q_ = 0.25 nm. The PMMA replica (Figure 6b) reproduces the original structure with the same roughness value, confirming high-fidelity replication. After application of the TASTE process (Figure 6c), the step features in both directions are significantly smoothened, resulting in a reduced roughness of S_q_ = 0.1 ± 0.05 nm. These results confirm the effectiveness of the TASTE process in selectively reducing nanoscale surface features, while also demonstrating the capability of AFM to resolve sub-nanometer topography and sub-100 pm roughness.

### 3.4. Pattern Transfer into PMMA

The linear slope geometry enables direct tracking of surface quality through successive replication steps. Replication into GMN PS90 preserves the slope geometry and roughness values over different length scales down to the nm scale.

Secondary replication into PMMA results in S_q_ = 4.1 ± 0.3 nm prior to post-processing.

**Figure 7:**
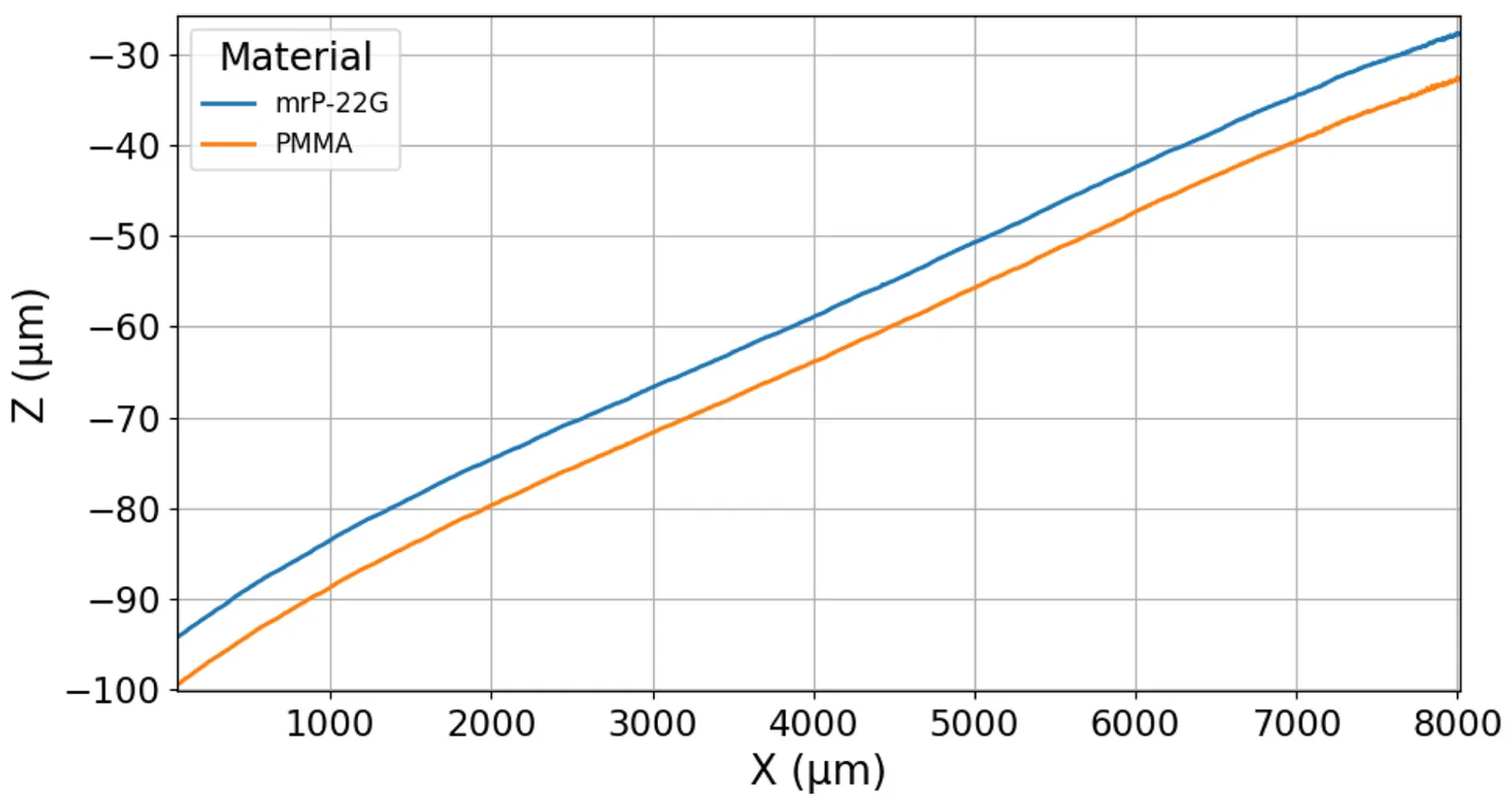
2D profile of linear slopes in mr-P 22G resist and PMMA. The mr-P 22G XP 2D profile was intentionally vertically offset (+6 µm).

The preservation of the slope geometry throughout the replication process up to the final PMMA replica is shown in Figure 7. For clarity, the mr-P 22G XP resist 2D profile was intentionally vertically offset to better visualize the overlap between the two profiles.

**Figure 8:**
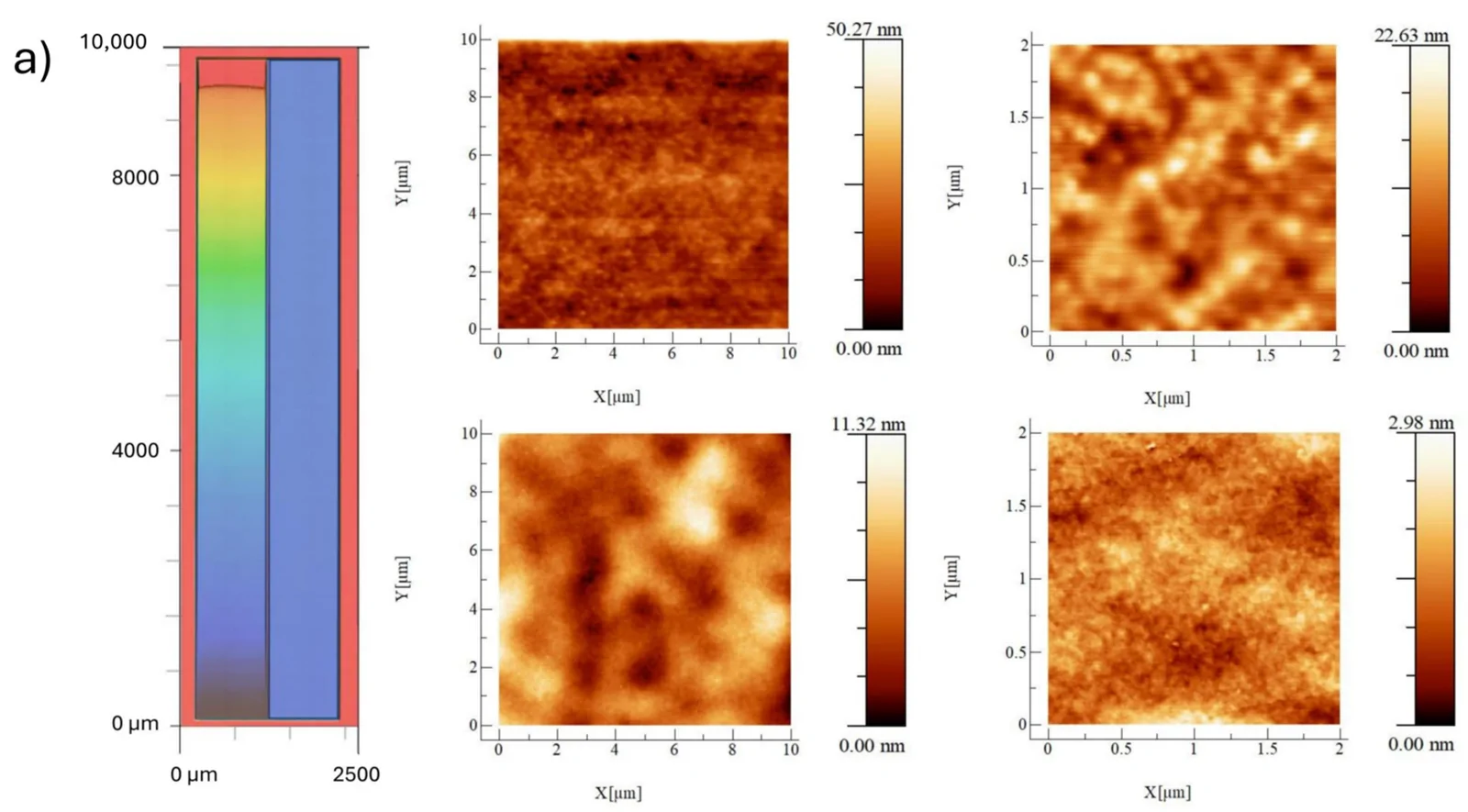
(**a**) LSCM measurements of linear slope in PMMA replicated from linear slopes fabricated in mr-P 22G XP on DWL 66^+^ using WM III, 256 GVs, and CI-over 40. (**b**,**d**) AFM image with scan of PMMA before TASTE; the scan area is 10 × 10 µm^2^ and 2 × 2 µm^2^, respectively. (**c**,**e**) AFM image with scan of PMMA after TASTE; the scan area is 10 × 10 µm^2^ and 2 × 2 µm^2^, respectively. The S_q_ values are indicated in the figures.

Application of TASTE to PMMA replicas results in a pronounced reduction of high-frequency roughness. LSCM measurements performed before and after TASTE showed no measurable change in the overall millimeter-scale slope profile. The modifications were therefore confined to the nanoscale surface topography and became apparent only in the AFM measurements. AFM measurements show a decrease from S_q_ = 4.1 ± 0.3 nm to S_q_ = 1.3 ± 0.1 nm for a scan area of 10 × 10 µm^2^ and a decrease from S_q_ = 3.9 ± 0.3 nm to S_q_ = 0.4 ± 0.1 nm for a scan area of 2 × 2 µm^2^, as shown in Figure 8. Because the two scan sizes probe different spatial-frequency ranges, the absolute values obtained from 10 × 10 µm^2^ and 2 × 2 µm^2^ areas are not directly compared; instead, the effect of TASTE is evaluated from the before/after reduction within each scan size.

The before- and after-TASTE AFM measurements were acquired in the central region of the same slope. However, because the measurement position was selected using the optical microscope and no fiducial markers were present on the slope, exact relocation of the same surface area was not possible. We estimate the positional uncertainty to be approximately ±100 µm. Therefore, the before/after comparison represents matched measurements from the same central slope region rather than the identical microscopic location.

### 3.5. Cross-Platform Validation of Intrinsic Roughness

AFM measurements were additionally performed on mr-P 22G XP resist exposed using the PICOMASTER system [38]. The comparison between the DWL 66+ and PICOMASTER was used primarily to distinguish deterministic, machine-related topographical artifacts from the nanoscale roughness. No measurable stitching artifacts or GV-induced waviness were observed in the PICOMASTER-exposed structures. Despite the absence of deterministic exposure artifacts, AFM measurements over comparable scan areas of 10 × 10 µm^2^ and 2 × 2 µm^2^ yield rms roughness values of 4.0 ± 0.5 nm and 4.1 ± 0.2 nm, respectively, consistent with those obtained on DWL-fabricated slopes (Section 3.2).

This result provides evidence that the observed nanoscale roughness is not limited by grayscale discretization or exposure strategy but instead represents an intrinsic material and process-dependent roughness floor of the novolak-based resist.

These findings further indicate that while different lithography platforms can significantly affect medium-spatial-frequency waviness, the high-frequency roughness remains largely invariant, reflecting fundamental material limitations.

## 4. Discussion

### 4.1. Dominant Roughness Sources at Each Stage

The comparison between the DWL 66+ and PICOMASTER was used primarily to distinguish deterministic, machine-related topographical artifacts from the residual nanoscale roughness. The quantitative evolution of roughness through the fabrication process chain is summarized in Table 2. While spin coating already results in a very low roughness of 1.1 ± 0.6 nm, the roughness increases due to the wet chemical development processing but stays at a relatively low level of <5 nm (artifacts and systematic errors excluded) that is seen as the fundamental limit of novolak. Replication preserves this roughness, which can be further reduced by thermal reflow to below 2 nm.

The results establish three key observations that form the basis of the Discussion: (i) deterministic waviness originates primarily from GV discretization and stitching; (ii) intrinsic nanoscale roughness is largely decoupled from grayscale exposure strategy; and (iii) post-processing (TASTE) enables selective suppression of stochastic roughness while preserving form. Observation (i) can be mitigated by optimizing the exposure process, observation (ii) shows the intrinsic limitations of the novolak resist, and observation (iii) is enabled by the material-inherent ability of low M_w_ PMMA to be smoothed down by thermal reflow.

The present study also highlights the complementary roles of LSCM and AFM for characterizing extended three-dimensional structures. LSCM enables rapid, non-contact measurement of form fidelity and waviness over millimeter-scale lengths and over bent (concave and convex) surface areas. However, when roughness approaches a few nanometers, LSCM measurements become limited by light diffraction and influenced by environmental vibrations, scanner noise, and stitching artifacts. Also, optical reflections and interferences in transparent samples could compromise measurements.

### 4.2. Separation of Form, Waviness, and Roughness in Linear Grayscale Slopes

The primary source of waviness originates from the discrete nature of grayscale DWL exposure. Stitching artifacts arising from the exposure strategy introduce additional periodic variations with characteristic lateral spacings of several tens of micrometers, but they were mitigated by optimized exposures. For a given slope height and length, the finite number of available gray values produces a quantized vertical profile with step-like height variations. The lateral step length observed in the LSCM measurements matches the expected gray-value discretization derived from the design parameters, confirming that this contribution is design-induced. Enhancing the number of GVs beyond the demonstrated 256 GVs and even beyond the 1’024 GVs possible with the DWL 66^+^ reduces the step length and height to values where smoothing becomes geometrically and physically favorable.

Because the spatial frequencies associated with roughness lie beyond the range resolved by LSCM, waviness and roughness must be treated separately when evaluating surface quality for optical applications. LSCM can, however, give an indication of advances toward lower roughness. In contrast, AFM measurements performed on localized regions reveal high-frequency stochastic height variations that are independent of the global slope geometry. These fluctuations represent the intrinsic surface roughness of the material and process.

### 4.3. Intrinsic Roughness and Roughness Transfer Through the Fabrication Chain

Cross-platform validation (Section 3.5) demonstrates that the observed roughness is independent of the lithography system and persists even in the absence of grayscale-induced waviness and stitching artifacts. This indicates that nanoscale roughness is not limited by the exposure strategy but represents an intrinsic material and process-dependent roughness floor of the novolak resist. This intrinsic roughness originates from nanoscale inhomogeneities within the novolak resist, including polymer chain packing, aggregation, and spatial variations in the distribution of PACs [22]. Such processes are known to produce nanometer-scale surface fluctuations with correlation lengths comparable to polymer molecular dimensions. The measured roughness values are consistent with previously reported results for thick positive-tone resists used in high-resolution lithography [39].

This length scale is consistent with the molecular dimensions of polymer resists used for high-resolution lithography, whose stochastic distribution limits the LER and surface roughness. One reason for this behavior is dependent on the nature of the polymer alone, including its monomer size, chain extension, mobility, and behavior when exposed (chain scission and removal of exposed material during development). Another reason is the scarcity of PAC molecules, their distributions within a volume element and the ability of the DNQ inhibitor molecules to produce phenolic clusters in the resist [23]. The first indicates how smooth a polymer surface can be; the second indicates how stochastic processes during exposure and development can lead to additional roughness. We exemplify this with two examples that analyze the fundamental limitations of LER: For the first, we use a molecular dynamics (MD) simulation from [40] on LER limitations in EBL, where chain scission is happening during the exposure of PMMA resist, providing insights into single-molecule modification and positioning. They found the typical structure of atomic-scale LER, i.e., the prominences of a molecular chain superimposed on an undulating molecular network. Such experiments demonstrate that single polymer chains can protrude from lines and therefore constitute, along with an undulating network, the minimum roughness of a line. LER simulations for M_w_ 1500 g·mol^−1^ and for 4000 g·mol^−1^ produced values of 0.6 nm and 0.7 nm, which are comparable to those that we achieved with TASTE and those which were measured by AFM. The authors claim that for these small values of M_w_, PMMA is liquid at room temperature, which would mean that VUV-exposed PMMA would already start reflowing at room temperature. Similar effects, i.e., of single polymer strands sticking out of the network of polymeric bulk, have been described by the same group for the molding of PMMA of different M_w_ in [41]. Whether this has a large influence on light scattering remains open.

For the limitations of LER in photoresists used in deep-UV-lithography, we adapt reasoning from Levinson [42]. Thick resists used in grayscale DWL are designed for low absorption, enabling exposures beyond 100 µm, which implies a sparse distribution of DNQ analogous to low photoacid generator (PAG) density in chemically amplified resists. At these scales, the size of resist molecules (e.g., phenolic components in novolak) becomes comparable to the targeted roughness and cannot be neglected. LER originates from the intrinsic inhomogeneity of molecule distribution within the resist. When PAGs are not polymer-bound, their distribution becomes uneven; even at high loading, typical spacings (~1.7 nm) set a fundamental roughness scale. In thick resists with low PAG concentration, this effect is amplified, as local solubility fluctuations during development lead to roughness on the order of a few nanometers (~4 nm). While the exact DNQ density in the novolak used is unknown, low loading suggests large intermolecular spacing. This supports a plausibility argument that the observed ~4 nm roughness limit originates from a combination of molecular size, aggregation effects, and spatial variations in solubility during development, which cannot be significantly reduced by thermal post-treatment alone.

The 60 min long development step also contributes to the final nanoscale surface morphology. AFM measurements of unexposed mr-P 22G XP subjected to the same 60 min development process show a roughness of approximately 4 nm, comparable to that measured on the developed grayscale slopes, while the spin-coated undeveloped resist exhibits a lower roughness. This comparison indicates that development itself contributes substantially to the characteristic few-nanometer surface roughness of the novolak resist. The roughness shows no significant dependence on slope angle or position along the slope, indicating that roughness is largely decoupled from macroscopic geometry. Grayscale exposure therefore primarily affects waviness, while roughness is governed by material and development processes at much smaller length scales. The measured roughness level (S_q_ ≈ 5 nm) thus represents the intrinsic roughness floor achievable with the present resist system. Replication of the slopes into GMN PS90 and PMMA demonstrates that nanoscale roughness is largely transferred through the fabrication chain.

### 4.4. Overcoming the Intrinsic Roughness Limit: Role of TASTE and Implications for X-Ray Optics

Processes such as SPEL have already reached roughness below the 4 nm limit identified as intrinsic roughness, which is mostly determined by the nature of the novolak used for photolithography [31]. Overcoming the intrinsic roughness limit requires materials different from novolak, with smaller molecules and strategies that enable surface smoothening after converting a 3D structure in novolak into an identical structure via pattern transfer (here replication). Although GMN-PS90, as a negative-tone resist that is crosslinked upon exposure, high-resolution capabilities proven in the process chain (Table 2), it is not suitable for post-processing via TASTE. Smoothing of novolak at a process temperature around the anticipated T_g_ of 90 °C, e.g., by thermal reflow, had only limited effect, since the polymer chains do not act as a liquid like PMMA molecules. TASTE has, in previous experiments, demonstrated a reduction in PMMA roughness from 22 nm to 5 nm [29]. Since most of the roughness was measured at structures with strong sidewall inclination, we anticipate that, even then, the actual roughness was lower. With the slope structures created by thermal NIL, AFM measurements demonstrate a reduction in roughness from 4.1 ± 0.3 nm to as low as S_q_ = 1.3 ± 0.1 nm (10 × 10 µm^2^) and 0.4 nm ± 0.1 nm (2 × 2 µm^2^), representing a transition from material-limited roughness to process-engineered surface quality. In the following, we take 1.3 nm as a confirmed value.

PMMA is expected to achieve significantly lower surface roughness due to its linear polymer structure, which enables smoother chain packing and reduces intrinsic roughness. Its excellent performance in high-resolution techniques such as electron-beam, X-ray lithography, and nanoimprint further demonstrates its capability for precise pattern transfer. Thermal imprinting in PMMA has been shown to replicate features as small as ~0.3 nm in height over a lateral length of ~5 nm, indicating an exceptional ability to preserve surface details with near-atomic fidelity. For a simple comparison, we choose a sphere that can be calculated, as done in [43,44], from the PMMA’s M_w_ and density. An M_w_ of 4 kg·mol^−1^ would be equivalent to 40 monomeric elements (100.12 g·mol^−1^ each) and would result in a sphere with a diameter 2.2 nm, in comparison to a diameter of 5.9 nm given for an unexposed M_w_ of 77 kg·mol^−1^. Such spheres can exceed the resolution demonstrated by thermal NIL. During molding, however, these structures are not rigid objects: polymer coils and even individual chains can deform under pressure and temperature. At the same time, single polymer chains sticking out of the surface may contribute to nanoscale roughness [40,41]. For microstructures, thermal reflow will lead to surface energy minimization, i.e., the amplitude of surface undulations will be reduced, eventually resulting in a completely flattened surface. In a review from 2019 [45], many of the questions about rounding, shape modification, and depth dependence were discussed in relation to the carried out experiments. Steps in staircase structures are typically rounded by localized transport of material from convex to concave areas, before the straight sections of stairs are smoothened out. Material transport occurs first between individual steps before longer-distance transport over several steps occurs. For the staircase structures within the slopes in this research, i.e., the 9.7 µm long, 59 nm high steps, this could lead to rounding of the steps while parts of the sections between steps remain straight. Higher reflow temperatures and longer process times could enhance polymer transport, but lateral flow may be limited to structures with a thickness close to the one that was modified by VUV (i.e., 200–400 nm). Since we did not see any straight sections after optimization of the writing strategy within the novolak, the main question concerns smoothing at the nanoscale. Here, with roughness in the nm range, exemplified by 0.26 nm high, 0.2 µm wide surface steps generated with sapphire molds, we anticipate that the geometry evolves during the process of smoothening as it does in microstructures; however, even the addition of single polymer chains can contribute to roughness. During VUV exposure, a range of polymer chain fragments are created, with a wide distribution of M_w_, making it possible that smaller entities with a higher ability to flow will be able to equilibrate surface undulations, while longer entities could still constitute roughness that adds to the surface undulations given by the polymer backbone. This is, in an exemplary way, demonstrated by the MD simulations in [41] for molding of nanoscale structures using PMMA with different M_w_ and in [46] for different mold sidewall profiles during demolding. More precise simulations, e.g., of the reflow process of molecular networks, are not yet available.

Thermal reflow, however, equilibrates surface undulations by self-optimization; therefore, smaller chain segments generated during exposure due to chain scission could contribute to reducing the attainable resolution and thus roughness. The current results, although demonstrating 1.3 nm roughness as the minimum, could in effect indicate that the actual roughness is much smaller. Moreover, even 0.4 nm roughness is currently not seen as the intrinsic roughness of PMMA. The value of 1.3 nm is already below the limitation at which we consider the LSCM suitable, and it might require further refinement of the AFM measurement parameters.

The difference between 4 nm and 1.3 nm is, however, significant for practical optical elements using EUV light. Scatter significantly reduces the contrast of a focal spot, which can be generated by reflecting light from a concave surface with grazing incidence at an angle of a few degrees. The minimum roughness of ~4 nm in the non-smoothened novolak surface would cause nearly 34% scattering at 80° grazing incidence for the EUV wavelength (according to the simplified formula (2) [47,48], where *TIS* is total integrated scattering, *P_0_* is incident light intensity, and *P_S_* is scattered light intensity, *σ* is rms roughness, λ is wavelength, *θ_i_* is the incident angle [35,36]). For a roughness of 1.3 nm measured over the 10 × 10 µm^2^ area, the scattering could be reduced to 4.3%; for a roughness of 0.4 nm measured over the 2 × 2 µm^2^ area, it could be reduced to 0.4%.

(2) $$TIS\equiv \frac{P_{S}}{P_{0}+P_{S}}=1-exp\left[-{\left(\frac{4\pi \sigma \cos\theta_{i}}{\lambda }\right)}^{2}\right]≅\frac{P_{S}}{P_{0}}≅{\left(\frac{4\pi \sigma \cos\theta_{i}}{\lambda }\right)}^{2}$$

## 5. Conclusions

This work demonstrates that the surface roughness of grayscale lithography structures is governed by two fundamentally different contributions: deterministic artifacts introduced during exposure and intrinsic nanoscale roughness arising from (resist) material properties and stochastic development processes. While exposure optimization effectively suppresses medium-spatial-frequency waviness, it does not significantly reduce the intrinsic roughness of novolak-based resists, which remains limited to ~4 nm. A significant reduction in roughness is achieved through a combination of replication into thermoplastic PMMA and post-processing via thermally activated selective topography equilibration (TASTE). This approach enables selective smoothing of high-frequency surface fluctuations, reducing roughness to the sub-2 nm regime while preserving the overall geometry of the structures. These results demonstrate that surface quality can be decoupled from the limitations of the initial resist by transferring the structures into materials with more favorable molecular properties and applying controlled thermal reflow.

Although a minimum roughness of ~4 nm in the non-smoothened novolak is too high for applications at the 13.5 nm EUV wavelength, the 1.3 nm roughness achieved so far would reduce the estimated scattering losses to below 5%. However, this value still remains considerably above the 0.2 nm roughness achieved by state-of-the-art glass polishing of EUV mirrors and is not suitable for any use with harder X-rays in the <10 nm range [48]. Further improvements below 1 nm roughness seem possible. In PMMA replicated from the sapphire staircase, an original roughness of 0.3 nm resulted in 0.1 nm after reflow. As this smoothing process is contactless and does not require mechanical polishing, it is not intrinsically limited to nanometer-scale roughness and may enable surface smoothness approaching the atomic scale.

The findings highlight the critical role of material selection and post-processing in achieving ultra-smooth polymer surfaces and provide a pathway toward meeting the strict roughness requirements of advanced optical applications.

## Figures and Tables

**Table 1:** Classification of the origins of defects affecting the LSCM and AFM measurements.

Class	Origin	Physical Meaning	Dominant Spatial Scale	Primary Instrument/Objective	Notes
Artifacts	Design- and machine-related	Stitching, gray-value (GV) discretization, multi-exposure effects	~10–50 µm	LSCM ×20 and ×50	Large field-of-view required to resolve stitching periodicity and quantization step length
Intrinsic roughness	Material/process	PAC statistics, polymer microstructure, stochastic dissolution, polymer flow	<1 µm	LSCM ×150 (optical limit) and AFM (nanoscale)	×150 provides highest optical vertical resolution; AFM used for quantitative nanoscale validation
Measurement noise	Metrology	Environmental vibrations, scanner jitter, stitching of optical images	Instrument-dependent	LSCM ×20, ×50, ×150	Present at all magnifications; more pronounced at ×150 due to higher axial sensitivity

**Table 2:** Quantitative evaluation of roughness through the fabrication steps from spin coating of novolak positive-tone resist to thermal reflow of the thermoplastic PMMA.

Process Step	Schematic	Material/Tool	Main Purpose	Dominant Surface Contribution	Roughness/Waviness (S_q_)	Mitigation/Optimization Strategy
Spin coating & soft bake	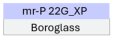	mr-P 22G XP	Define resist thickness (≈100 µm)	Intrinsic material roughness	1.1 ± 0.6 nm	Degassing, controlled bake ramp
Grayscale DWL exposure	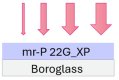	DWL66^+^	Encode linear slope geometry	Dose discretization → waviness	1.8 ± 0.2 nm	Multi-pass/N-over exposure
Development	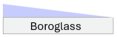	AZ726 MIF developer	Reveal 3D topography	Stochastic development noise	4.0 ± 0.2 nm	Dose calibration, dev. time control
Resist reflow	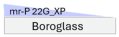	mr-P 22G XP @80 °C	Surface smoothing	Roughness and waviness	4.0 ± 0.2 nm	—
Replication	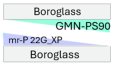	GMN PS90 with UV-imprint	Enable slope replication	Replication defects	4.2 ± 0.3 nm	Optimized imprint parameters
Secondary replication	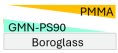	PMMA by thermal imprint	TASTE-compatible material	Material roughness increase	4.1 ± 0.3 nm	Controlled imprint conditions
TASTE process	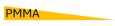	Thermal equilibration	Reduce high-frequency roughness	Depth-selective (~200 nm) roughness reduction	1.3 ± 0.1 nm	Time–temperature tuning

## Data Availability

The raw data supporting the conclusions of this article will be made available by the authors on request.

## Supplementary Materials

The following supporting information can be downloaded at: https://www.mdpi.com/article/10.3390/polym18172148/s1, Figure S1. RMS roughness values for the gray-tone photoresist mr-P22G_XP at different manufacturing stages: (a) spin-coated and soft-baked, RMS roughness = 1.1 ± 0.6 nm; (b) exposed, RMS roughness = 1.8 ± 0.2 nm, (c) developed without prior exposure, RMS roughness = 2.3 nm and (d) exposed and developed, RMS roughness = 4.0 ± 0.2 nm nm. Globular surface structures forming during development are clearly visible. A 71% roughness increase was additionally observed between spin coated/soft baked and exposed resist. High-resolution scans (c) and (d) were acquired with a Bruker Multimode AFM in tapping mode in air using identical cantilevers (RTESPA-150). Figure S2. AFM roughness measurements acquired prior to resist reflow. (a) 100 × 100 µm^2^ scan showing both stitching artefacts (vertical lines, spacing ~30 µm) and grayscale-value (GV) steps (horizontal lines, spacing ~9.7 µm), yielding an aggregate RMS roughness of 55 nm. (b) 50 × 50 µm^2^ shows a close-up of the GVs in between two stitching artifacts. The RMS roughness acquired between two stitching artefacts, showing only GV-step contributions. The RMS roughness measured across adjacent GV steps is 8 nm. (c) 1 × 1 µm^2^ scan acquired within one GV plateau, excluding stitching and discretization effects. The measured RMS roughness is 4.5 nm. Figure S3. AFM topography images acquired at different positions along the same slope profile prior to resist reflow. (a) Upper section (beginning of the slope exposed with low dose), where no distinct GV steps are observed. (b) Middle section (~60 µm depth) and (c) deepest section (~100 µm depth, exposed with highest dose), where discrete GV steps become visible.

## Abbreviations

The following abbreviations are used in this manuscript:

AFM	Atomic Force Microscopy
ASL	Anti-Sticking Layer
DNQ	Diazonaphthoquinone
EUV	Extreme Ultraviolet
GV	Gray Value
LCSM	Laser Confocal Scanning Microscope
LER	Line-Edge Roughness
PAC	Photoactive Compound
PAG	Photoacid Generator
TASTE	Thermally Activated Selective Topography Equilibration
TIS	Total Integrated Scattering
