# Supplementary material for: Nanoscale Roughness in Ultra-Thick Resists by Laser-Scanning Grayscale Direct-Write Lithography and Surface Smoothing
Source: Polymers (Basel). 2026 Sep 2;18(17):2148. doi: 10.3390/polym18172148 (PMC13567985; doi:10.3390/polym18172148)
Supplement: Supplementary file 1 [file polymers-18-02148-s001.zip › polymers-4430758-supplementary.pdf]

Supporting Information

This document provides the supporting information for the manuscript entitled *"Nanoscale roughness in ultra-thick resists by laser-scanning grayscale direct-write lithography and surface smoothening"*.

The figures included in this Supporting Information provide additional insight into the origin and evolution of surface roughness during grayscale lithography. In particular, they illustrate the contributions arising from development, grayscale discretization, and stitching artefacts prior to the resist reflow step. Since the main manuscript focuses on the surface quality of the final structures after process optimization and thermal reflow, only post-reflow roughness values are discussed in the main text. The measurements presented here are intended to support the interpretation of the roughness mechanisms and to demonstrate the effectiveness of the resist smoothing process.
Figure S1 illustrates the evolution of roughness through the different fabrication stages, from spin coating to development. These measurements help identify the stage at which the roughness reaches its final value and provide insight into the contribution of the development process to surface morphology.
Figures S2 and **Figure S3** present AFM measurements acquired prior to the resist reflow step. The structures were fabricated using 1024 grayscale values (GVs) and a CI-over 10 multi-exposure strategy. At this stage of the process, stitching artefacts arising from the exposure strategy are visible (as discussed in Section 3.1) and the discrete grayscale-level steps remain visible on the surface. Consequently, the measured roughness includes contributions also from stitching boundaries and grayscale discretization. The subsequent resist reflow process smoothens the grayscale-level steps. Because the objective of the work is to demonstrate the achievable roughness of the final structures after process optimization, the manuscript reports only resist post-reflow measurements.

The measurements reported in the Supporting Information were acquired using a different AFM system than some of those presented in the main manuscript. However, identical roughness evaluation procedures were applied, and the observed roughness values and trends are fully consistent with those reported in the main text. The supporting measurements are therefore considered complementary, representative and valid for the interpretation of the fabrication process.
The following AFM roughness measurements were acquired at multiple positions along the slope profile using an NT-MDT NTEGRA (NT-MDT Spectrum Instruments) operating in tapping mode in air. Silicon cantilevers (RTESPA-150, Bruker) with a nominal resonance frequency of 150 kHz, a spring constant of 5 N/m, and a tip radius of ~8 nm were employed.

S1. Roughness Measurements of Spin-Coated, Unexposed, and Developed Resist

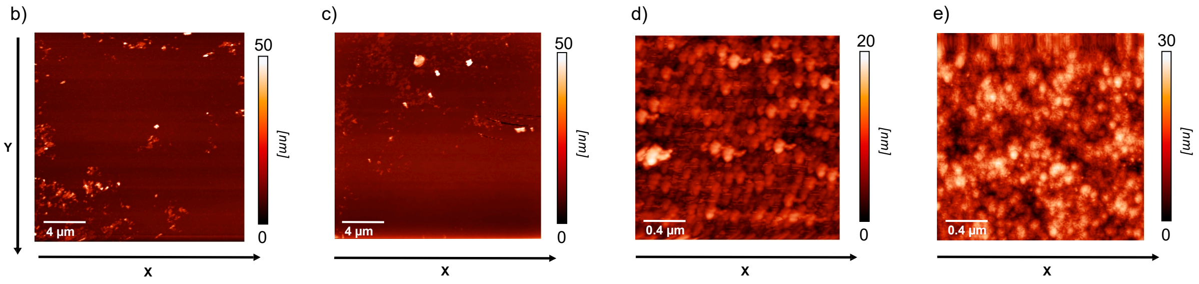

Figure S1. RMS roughness values for the gray-tone photoresist mr-P22G_XP at different manufacturing stages: (a) spin-coated and soft-baked, RMS roughness = 1.1 ± 0.6 nm; (b) exposed, RMS roughness = 1.8 ± 0.2 nm, (c) developed without prior exposure, RMS roughness = 2.3 nm and (d) exposed and developed, RMS roughness = 4.0 ± 0.2 nm nm. Globular surface structures forming during development are clearly visible. A 71% roughness increase was additionally observed between spin coated/soft baked and exposed resist. High-resolution scans (c)

and (d) were acquired with a Bruker Multimode AFM in tapping mode in air using identical cantilevers (RTESPA-150).

S2. Surface Roughness of Slope Profile before Resist Reflow

To distinguish between contributions of fabrication process and intrinsic surface roughness of the resist, AFM measurements were performed at different locations and over different scan areas. Measurements performed across stitching boundaries yielded an RMS roughness of 55 nm (Figure S2a), as reported in Section 3.1 Figure 4. When AFM scans were acquired across two grayscale levels (across a step), while avoiding stitching boundaries (Figure S2b), the measured RMS roughness decreased to 8 nm. This value still includes contributions from the discrete height steps. To evaluate the roughness of the exposed resist, additional scans were acquired within a single grayscale step. Under these conditions, the RMS roughness was found to be below 4.5 nm. Following the resist thermal reflow process, the discrete grayscale steps are smoothed, resulting in a continuous surface profile. After the reflow, the roughness measured over the entire slope approaches the roughness measured within an individual plateau, yielding a homogeneous roughness below 4.5 nm across the full structure.

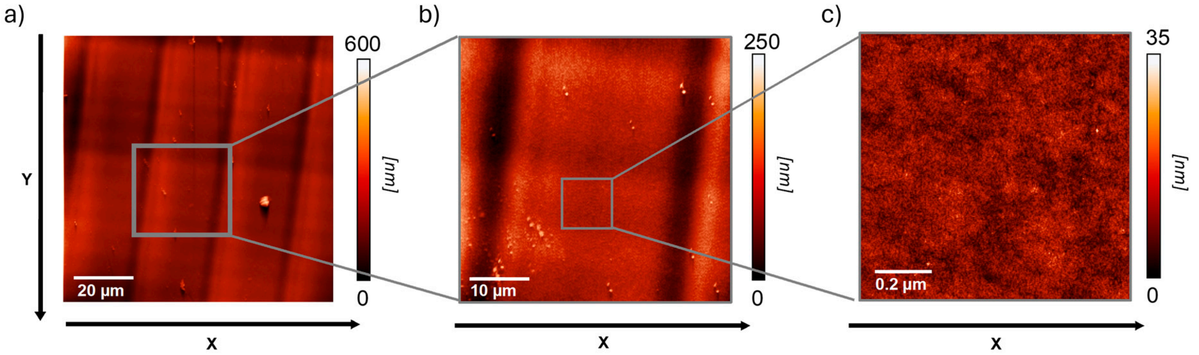

Figure S2. AFM roughness measurements acquired prior to resist reflow. (a) 100 × 100 µm² scan showing both stitching artefacts (vertical lines, spacing ~30 µm) and grayscale-value (GV) steps (horizontal lines, spacing ~9.7 µm), yielding an aggregate RMS roughness of 55 nm. (b) 50 × 50 µm² shows a close-up of the GVs in between two stitching artifacts. The RMS roughness acquired between two stitching artefacts, showing only GV-step contributions. The RMS roughness measured across adjacent GV steps is 8 nm. (c) 1 × 1 µm² scan acquired within one GV plateau, excluding stitching and discretization effects. The measured RMS roughness is 4.5 nm.

S3. GV height difference along the slope

To investigate the contribution of grayscale discretization to the surface morphology, AFM measurements were acquired at different locations along the same slope profile prior to resist reflow. Due to the non-ideal linearity of the fabricated slope, the local slope angle varies along its length. Near the beginning of the slope, the height difference between adjacent grayscale values (GVs) is sufficiently small that individual GV steps are not distinguishable in the AFM images. Further along the slope, where the local slope angle increases, step heights of 12.3 ± 2.0 nm (Figure S3b) and 20.3 ± 2.2 nm (Figure S3c) were measured at approximately 37.5 µm and 74 µm developed depth, respectively.. Following the thermal resist reflow process, the GV steps are smoothed, resulting in a continuous surface profile (see Section 3.2).

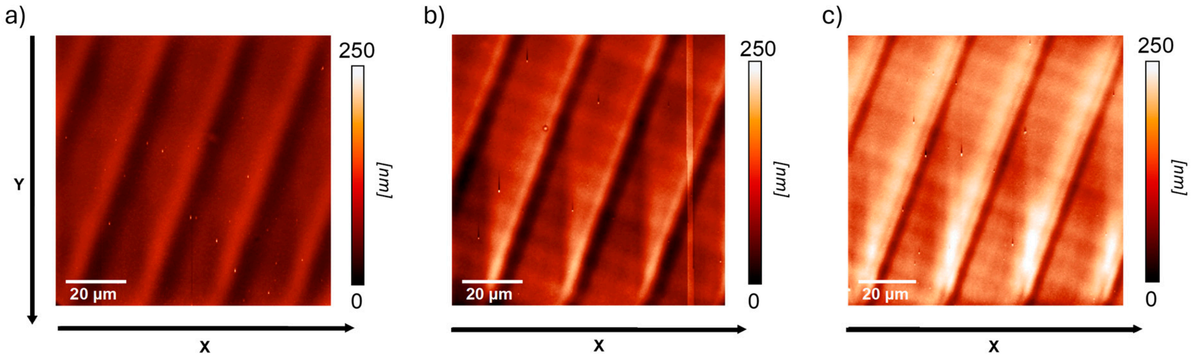

Figure S3. AFM topography images acquired at different positions along the same slope profile prior to resist reflow. (a) Upper section (beginning of the slope exposed with low dose), where no distinct GV steps are observed. (b) Middle section (~60 µm depth) and (c) deepest section (~100 µm depth, exposed with highest dose), where discrete GV steps become visible.
